# Circadian mechanisms in heart failure: Cell-specific regulation and chrono-medicine

**DOI:** 10.1016/j.cellin.2026.100363

**Published:** 2026-09-05

**Authors:** Shuting Cheng, Runchao Zhou, Zhihui Zhang, Min-Dian Li

**Affiliations:** a NHC Key Laboratory of Chronobiology (Sichuan University), West China School of Basic Medical Sciences & Forensic Medicine, Sichuan University, Chengdu, Sichuan, 610041, China; b Department of Cardiovascular Medicine, Southwest Hospital, Army Medical University, Chongqing, 400038, China; c Key Laboratory of Chronobiology and Cardiometabolic Disease, Chongqing Education Commission of China, Chongqing, 400038, China; d Key Laboratory of Geriatric Cardiovascular and Cerebrovascular Disease (Army Medical University), Ministry of Education of China, Chongqing, 400038, China

**Keywords:** Circadian rhythm, Heart failure, Metabolism, Fibrosis, Chronotherapy

## Abstract

Emerging evidence indicates that circadian rhythms profoundly influence the pathophysiology of heart failure (HF). The circadian clock system governs myocardial functions via synchronized actions from multiple cell types involving cardiomyocytes and non-cardiomyocytes. This review summarizes state-of-the-art advances on the cardiovascular circadian system and provides a perspective on time-of-day effects in the pathophysiology of HF, including energy metabolism, tissue inflammation and fibrosis. In particular, we focus on cell-specific circadian regulation and synthesize how circadian clocks across cardiomyocytes, vascular cells, fibroblasts, and adipocytes interact to regulate the pathophysiology of HF. Based on this framework, we discuss how synchronizing internal and external time cues via clock-modulating compounds and/or time-restricted eating (TRE) may prevent HF or facilitate rehabilitation. Together, this Review provides a timely synthesis of current understanding of HF as a function of biological timing and highlights potential chronotherapeutic strategies.

## 1 Introduction

Heart failure (HF) is a clinical syndrome resulting from impaired cardiac structure or function (Heidenreich et al., 2022), affecting an estimated 55.50 million individuals worldwide (Ran et al., 2025). HF is classified by left ventricular ejection fraction into HF with reduced ejection fraction (HFrEF), HF with mildly reduced ejection fraction (HFmrEF), and HF with preserved ejection fraction (HFpEF). In HFrEF, neurohormonal activation drives cardiac remodeling, and therapies targeting the renin-angiotensin-aldosterone system and sympathetic nervous system, together with sodium-glucose co-transporter 2 (SGLT2) inhibitors, have substantially improved survival (McDonagh et al., 2021; Hartupee & Mann, 2017). HFpEF, which is steadily increasing and projected to become the dominant phenotype, involves metabolic comorbidities and systemic inflammation that impair diastolic function; no unifying mechanism has been established (Hamo et al., 2024). Although SGLT2 inhibitors and GLP-1 receptor agonists have shown benefit in HFpEF (Kosiborod et al., 2023; Solomon et al., 2022), 1-year mortality remains 15%–30% and 5-year mortality 50%–75% (Stolfo et al., 2022), underscoring the need for deeper mechanistic investigation.

Time-of-day influences the onset of HF-related pathological events, including myocardial infarction, ventricular arrhythmias, and sudden cardiac death, with a higher incidence in the early morning (Delisle et al., 2024). This temporal vulnerability is mediated by the cardiomyocyte-intrinsic circadian clock (Tikhomirov et al., 2024): in mice, ischemia at different circadian phases produces significantly different infarct sizes, and cardiomyocyte-specific clock mutation abolishes this time-of-day dependence (Durgan et al., 2010); clinically, patients undergoing cardiac surgery in the afternoon sustain less perioperative myocardial injury than those operated in the morning (Montaigne et al., 2018).

Circadian gating also affects the inflammatory response. The tolerance of myocardium to ischemia-reperfusion injury is poor during the early morning, coinciding with the peak of pro-inflammatory factors secreted by activated platelets (Eckle et al., 2024), and sustained inflammation contributes to cardiomyocyte dysfunction, adverse remodeling, and contractile impairment (Alcaide et al., 2024). Circadian disruption further exacerbates chronic HF by impairing cardiac metabolic flexibility, reducing mitochondrial oxidative efficiency, and engaging regulated cell-death programs including necroptosis, thereby contributing to inflammatory amplification and adverse remodeling (Actis Dato et al., 2024; Dabravolski et al., 2025; Xu et al., 2025).

Moreover, circadian disruption under constant light facilitates cardiac remodeling with lower systolic function, larger left ventricular volume, and aggravated cardiac fibrosis after myocardial infarction via sympathetic activation (Wang et al., 2021). Notably, in patients with established HFrEF, peripheral cardiac clocks remain functional, whereas central clock output is markedly dampened (Crnko et al., 2023), underscoring the need to elucidate how the circadian clock regulates HF-relevant processes at the level of individual cardiac cell types.

The major cardiac cell types, including cardiomyocytes, cardiac fibroblasts, endothelial cells (ECs), vascular smooth muscle cells (SMCs), and resident immune cells, each harbor functional circadian clocks (Crnko et al., 2019; Scheiermann et al., 2018). Single-nucleus RNA sequencing (snRNA-seq) and single-cell RNA sequencing (scRNA-seq) of the healthy adult human heart have yielded a comprehensive atlas of 11 major cell types (Litvinukova et al., 2020); their distributions, proportions, and marker genes are listed in Table 1. Murine hearts across ages were also profiled in the Tabula Muris Senis multi-organ aging atlas, from which we derived age-related proportions of 11 cardiac cell types (Table 2) (Tabula Muris, 2020). Marker gene panels were referenced from Skelly et al., DeLaughter et al., and Hulin et al. (DeLaughter et al., 2016; Hulin et al., 2019; Skelly et al., 2018). We identified HF-relevant cardiac cell types, reviewed their circadian regulation, and assessed how clock disruption contributes to HF progression. Together, this single-cell approach provides a mechanistic framework for understanding circadian regulation of HF pathophysiology and a rationale for chronotherapeutic intervention.

**Table 1:** Major cell types and proportions in adult heart.

Cell type	Atrial (%)	Ventricular (%)	Marker genes
Ventricular cardiomyocytes	0	49.2	MYH7, MYL2, FHL2
Atrial cardiomyocytes	30.1	0	NPPA, MYL7, MYL4
Fibroblasts	24.3	15.1	DCN, GSN, PDGFRA
Pericytes	12.9	19	RGS5, ABCC9, KCNJ8
Endothelial cells	12.2	7.8	VWF, PECAM1, CDH5
Myeloid cells	5.5	3.7	CD14, C1QA, CD68
Smooth muscle cells	4.2	2.2	MYH11, TAGLN, ACTA2
Lymphoid cells	4.9	1.6	CD8A, IL7R, CD40LG
Neural cells	2.3	0.6	PLP1, NRXN1, NRXN3
Adipocytes	3.1	0.7	GPAM, FASN, LEP
Mesothelial cells	0.6	0	MSLN, WT1, BNC1

**Note:** Values for human ventricular fibroblasts (15.1%) are from Supplementary Table 2 of a previous study (Litvinukova et al., 2020).

**Table 2:** Changes in mouse heart cell proportions with age.

Cell type	3 months (%)	18 months (%)	24 months (%)	Marker genes
Fibroblasts	47.78	37.79	25.68	Lamc1, Pcsk6, Pdgfra, Entpd2, Dpep1, Adamts5, Medag, Ms4a4d, Lamb1, Tcf21, Dkk3, Tbx20, Wif1, Frzb, Meox1, Prg4, Abi3bp, Pdgfrl, Mdk, Gstm5
Endothelial cells (coronary)	26.60	22.33	33.19	Ly6c1, Egfl7, Gpihbp1, Cdh5, Mgll, Slc9a3r2, Emcn, Kdr, Pecam1, Rgcc
Monocytes	11.05	16.77	14.03	Itgam, Cd14, Csf1r, Fcer1g, Ccr2, Ly6c2, Cx3cr1, Lgals3, Cd68, F13a1
Atrial myocytes	3.59	4.88	4.52	Nppa, Myl7, Myl4, Sln, Gja5, Nppb, Tbx5, Hey1, Nr2f2, Kcna5
Endocardial cells	3.70	2.29	5.15	Npr3, Hapln1, Nfatc1, Sox17, Emcn, Fabp4, Nrg1, Bmp4, Cdh5, Pecam1
Valve cells	3.56	2.00	3.64	Twist1, Tbx20, Msx1, Msx2, Postn, Fbn1, Col1a1, Col3a1, Vcan, Acta2
Smooth muscle cells	1.47	0.39	0.94	Tagln, Mustn1, Myh11, Mylk, Pcp4l1, Sncg, Lmod1, Des, Pln, Nrip2
Ventricular myocytes	1.04	0.00	3.17	Myl2, Myh7, Fhl2, Irx4, Hey2, Actc1, Tnni3, Cacna1c, Ryr2, Gja1
T cells	0.83	4.53	2.10	Cd3g, Cd3d, Lat, Cd3e, Skap1, Il7r, Lef1, Cd247, Tcf7, Itk
B cells	0.34	6.19	6.84	Cd79a, Ly6d, Cd79b, H2-DMb2, Ms4a1, H2-Ob, Fcmr, Ccr7, Bank1, Cd55
Macrophages	0.05	2.83	0.72	Dab2, Adgre1, Mgl2, Mrc1, Hpgd, P2ry6, C3ar1, F13a1, Maf, Ms4a7

**Note:** No ventricular myocytes were captured in the TMS 18-month heart sample (2 051 total cells sequenced); the value is therefore reported as 0.00%.

## 2 The circadian clock in the cardiovascular system

Circadian rhythms are ∼24 h cycles orchestrated by endogenous molecular clocks (Takahashi, 2017). The circadian system comprises a central clock and peripheral clocks organized hierarchically (Mohawk et al., 2012). The central clock, located in the suprachiasmatic nucleus (SCN), is entrained by light/dark cycles (Welsh et al., 2010). It coordinates peripheral clocks via neural and humoral cues (Koronowski & Sassone-Corsi, 2021); the heart and other key peripheral tissues possess cell-autonomous molecular clocks. Nutrient availability entrains peripheral clocks in metabolic tissues, while mechanical and hemodynamic signals entrain cardiac clocks (Takahashi, 2017; Zhang et al., 2020). At the core of this circadian machinery in cardiac cells lie interlocked transcriptional-translational feedback loops (TTFLs) (Takahashi, 2017) (Fig. 1).

(1) **The core negative feedback loop**: The primary loop centers on the basic helix-loop-helix PER-ARNT-SIM (bHLH-PAS) transcription factors Brain and muscle ARNT-like 1 (BMAL1) and Circadian locomotor output cycles kaput (CLOCK). BMAL1 and CLOCK heterodimerize and initiate the expression of clock-controlled genes by binding to E-box elements in the enhancer/promoter regions. Key targets include Period (*PER1*, *PER2*) and Cryptochrome (*CRY1*, *CRY2*) genes.
(2) **Negative feedback**: PER and CRY proteins form complexes in the cytoplasm, translocate back into the nucleus, and gradually inhibit the transactivation activity of the BMAL1:CLOCK complex, thus repressing the transcription of *PER/CRY* genes.
(3) **The stabilizing loop**: A secondary loop reinforces the core cycle and defines its periodicity. BMAL1:CLOCK also activates the transcription of the nuclear receptors *Rev-erbα* and *Rev-erbβ*, as well as RAR-related orphan receptors (*RORα*/*β*/*γ*). REV-ERBs act as potent transcriptional repressors by competing with activating RORs for binding to ROR response elements (ROREs) in the promoters of *Bmal1*, Nuclear factor interleukin 3 regulated (*Nfil3*), and *Clock.* This accessory loop consisting of ROR (activator)/REV-ERB (repressor) stabilizes the amplitude and timing of core clock gene expression. The D site-binding protein (*Dbp*), another BMAL1:CLOCK target, also contributes to this regulation and influences output gene expression. Detailed discussion of these mechanisms can be found elsewhere (Koronowski & Sassone-Corsi, 2021; Takahashi, 2017).

**Fig. 1:**
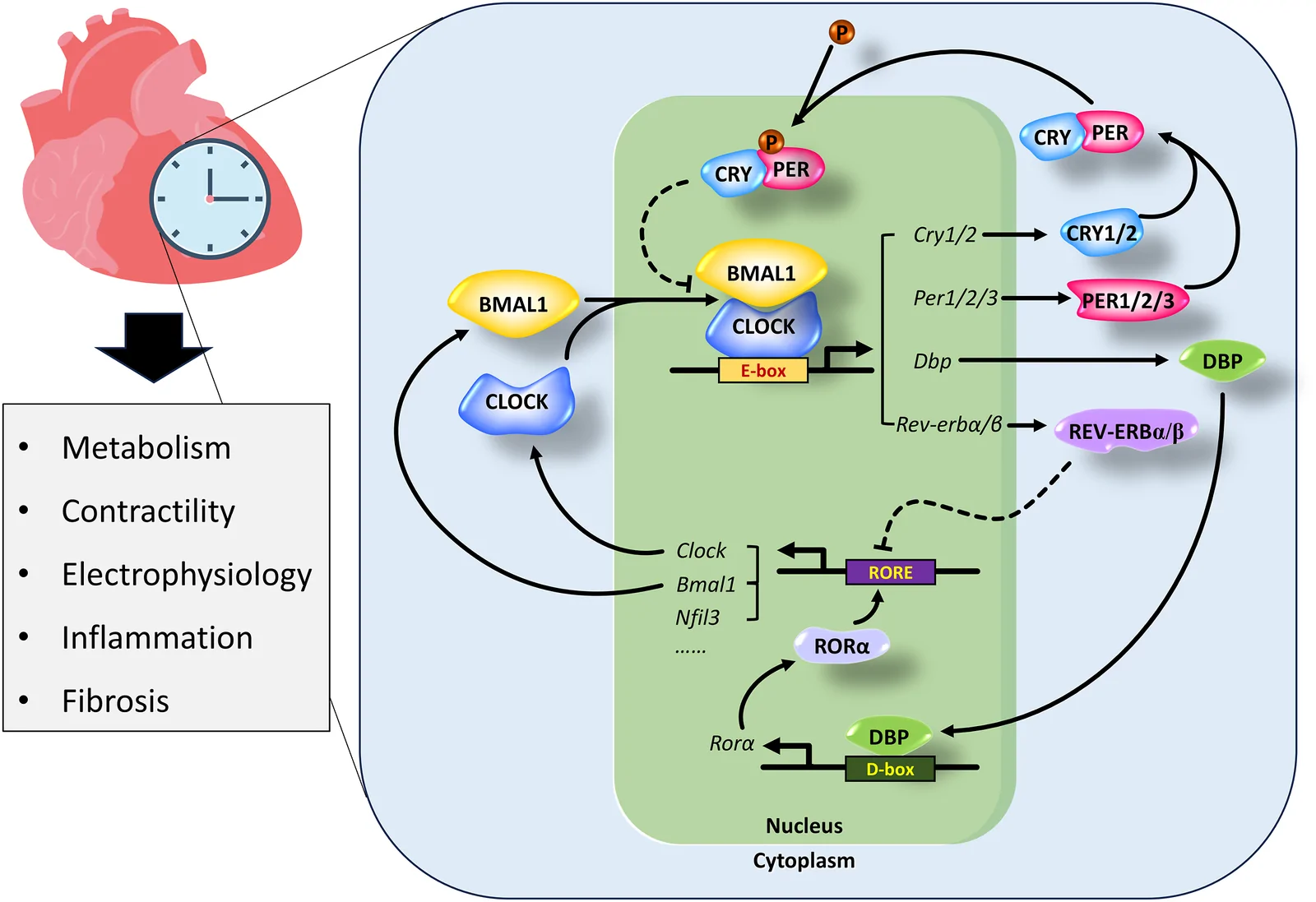
**The molecular circadian clock in the heart.** The core molecular circadian clock in cardiac cells operates through interlocked transcriptional-translational feedback loops (TTFLs). The positive transcriptional arm is driven by the BMAL1:CLOCK heterodimer, which binds to E-box elements in the promoters of target genes to drive transcription of the core clock components Period (*Per1*/*2*), Cryptochrome (*Cry1*/*2*), RAR-related orphan receptors (*Rorα*/*β*/*γ*), D-site binding protein (*Dbp*) and *Rev-erbα*/*β*. The negative feedback arm operates as PER and CRY proteins form complexes in the cytoplasm, translocate into the nucleus, and progressively inhibit the transactivation activity of the BMAL1:CLOCK heterodimer, thereby repressing their own transcription. The stabilizing loop involves REV-ERBα/β proteins, which act as transcriptional repressors by competing with activating RORs for binding to ROR response elements (ROREs) in the promoters of *Bmal1* and *Nfil3* (and to a lesser extent *Clock*). This antagonistic ROR/REV-ERB relationship tightly regulates the amplitude and timing of core clock gene expression, particularly *Bmal1*. REV-ERB-mediated repression of *Nfil3* further modulates the core clock output. Together, these interconnected loops generate circadian (∼24 h) rhythmicity in gene expression that coordinates essential cardiac processes including metabolism, contractility, electrophysiology, inflammation, and fibrosis. BMAL1, Brain and muscle ARNT-like 1; CLOCK, Circadian locomotor output cycles kaput; PER, Period; CRY, Cryptochrome; ROR, RAR-related orphan receptor; REV-ERB, Reverse-Erb; DBP, D-box binding protein; NFIL3, Nuclear factor interleukin 3 regulated.

While the core clock genes are shared across tissues, the clock-regulated biological processes are tissue-specific (Takahashi, 2017). This oscillator is active in the major cardiac cell types, including cardiomyocytes, ECs, vascular SMCs, fibroblasts and resident immune cells (Crnko et al., 2019). The rhythmic expression of clock genes and their protein products within these cells provides the molecular foundation for circadian regulation of crucial cardiac processes relevant to HF, including metabolism, contractility, electrophysiology, inflammation, and fibrosis.

## 3 Circadian disruption in HF

The circadian clock temporally organizes cellular processes that maintain cardiac homeostasis. Disruption of this temporal organization amplifies HF-relevant pathology, including myocardial injury, metabolic dysfunction, inflammation and fibrosis.

### 3.1 Myocardial injury and inflammation

The heart's vulnerability to damage, its inflammatory activity after injury, and its metabolic efficiency all exhibit daily fluctuations under the control of the circadian clock. Disruption of these rhythms increases HF susceptibility and accelerates its progression. A key clinical manifestation of this rhythmic vulnerability is the well-documented morning surge in cardiac events (Fig. 2, blue pathway). Consistent with this epidemiological pattern (Rabinovich-Nikitin et al., 2019), Durgan et al. demonstrated that myocardial I/R tolerance is time-of-day dependent in mice, with the worst outcomes occurring at the rest-to-active transition, and that this rhythm is driven by the cardiomyocyte circadian clock (Durgan et al., 2010).

**Fig. 2:**
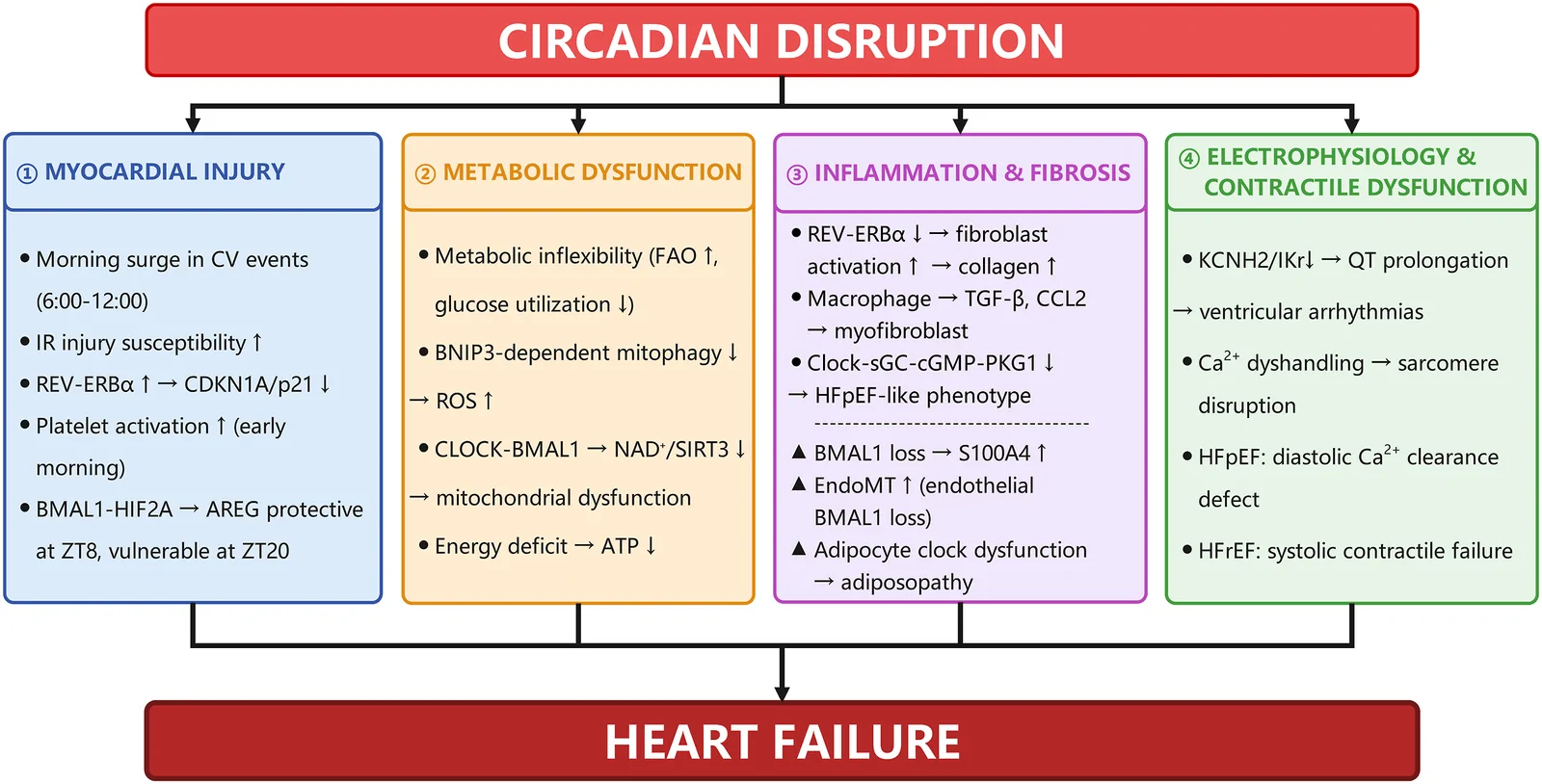
**Circadian disruption as a driver of heart failure pathophysiology.** This schematic illustrates the hierarchical mechanistic framework by which circadian clock dysfunction promotes the progression of heart failure (HF). The central node represents circadian disruption, characterized by BMAL1 downregulation, Clock dysfunction, and central-peripheral decoupling. This central perturbation propagates through four interconnected pathological pathways: (1) Myocardial injury (blue): Circadian disruption exacerbates ischemia-reperfusion injury susceptibility, particularly during the morning hours (6:00-12:00) when cardiovascular events peak. Elevated REV-ERBα represses CDKN1A/p21 expression, compromising cardiomyocyte survival. Platelet activation peaks in early morning, promoting pro-inflammatory microenvironment. The BMAL1-HIF2A heterodimer rhythmically induces amphiregulin (AREG), conferring cardioprotection at ZT8 (rest phase) but vulnerability at ZT20 (active phase). (2) Metabolic dysfunction (orange): BMAL1 deficiency leads to metabolic inflexibility with sustained fatty acid oxidation and impaired glucose utilization via disrupted AKT/GSK3β signaling, accompanied by defective BNIP3-dependent mitophagy, suppressed respiratory complex activities, and increased ROS generation. The CLOCK-BMAL1-driven NAD^+^/SIRT3 axis is impaired, further compromising mitochondrial oxidative metabolism. The resulting energetic deficit undermines cardiac ATP demands. (3) Inflammation and fibrosis (purple): REV-ERBα restricts pathological fibroblast activation and collagen secretion; its loss impairs this inhibitory brake. Macrophage-derived TGF-β and CCL2 drive fibroblast activation and myofibroblast differentiation. Chronic jet lag reproduces an HFpEF-like phenotype through Clock-sGC-cGMP-PKG1 pathway suppression. Proposed interactions (triangle bullets in the figure): BMAL1 loss in myocardial stromal fibroblasts derepresses S100A4; endothelial BMAL1 deficiency promotes EndoMT-like changes; and adipocyte clock dysfunction contributes to adipose inflammation and adverse remodeling. (4) Electrophysiology and contractile dysfunction (green): BMAL1 ablation downregulates key repolarizing K^+^ channels (most notably Kcnh2/IKr), leading to QT prolongation and heightened risk of malignant ventricular arrhythmias. Disordered Ca^2+^ cycling and disorganized sarcomeric structures impair force generation: HFpEF is characterized by a diastolic Ca^2+^ clearance defect, whereas HFrEF involves systolic contractile failure. The convergence of electrophysiological, contractile, and metabolic derangements culminates in unstable rhythms, diminished cardiac output, and heightened risk of sudden cardiac death. Solid bullets (☆) denote established mechanisms; triangle bullets (▲) denote proposed interactions requiring further validation.

The mechanistic underpinnings of this time-of-day effect involve the core clock regulator REV-ERBα. Elevated REV-ERBα expression (peaking during the active phase in mammals) paradoxically promotes cardiomyocyte death during hypoxia-reoxygenation stress. This detrimental effect arises because high REV-ERBα levels directly repress the transcription of the protective survival factor CDKN1A/p21 (Fig. 2, blue pathway), leaving cardiomyocytes more vulnerable to injury, providing a molecular basis for the morning vulnerability of ischemic myocardial injury (Montaigne et al., 2018). Moreover, this cardiac vulnerability is amplified by the circadian activation of platelets, which is driven by the platelet-intrinsic clock and peaks during the early morning (Shi et al., 2022), fostering a pro-inflammatory microenvironment that promotes infarction and amplifies post-injury damage (Eckle et al., 2024).

These findings suggest that myocardial injury is amplified around the sleep-to-wake or rest-to-active transition, broadly corresponding to the early morning in humans. This phase-related vulnerability may arise from the convergence of lower ischemic tolerance, pro-death signaling, and platelet inflammatory activation, although the relative contribution of each mechanism remains to be clarified.

### 3.2 Metabolic dysregulation

The heart's energy metabolism operates with robust circadian rhythms essential for efficient fuel utilization and energy production (Wang et al., 2025). Substrate utilization is precisely gated by circadian clocks: glucose oxidation dominates active phases via PER2-mediated PFK activation, while fatty acid oxidation prevails at rest through REV-ERBα/β regulation of CPT1A/ECH1 (Zhang & Jain, 2021). At the core of this metabolic rhythmicity, the CLOCK-BMAL1-driven NAD^+^ biosynthesis cycle sustains SIRT3-dependent mitochondrial oxidative metabolism (Peek et al., 2013), while circadian control of DRP1 activity regulates mitochondrial dynamics and bioenergetics (Schmitt et al., 2018). This rhythmic metabolic program is critically impaired in ischemic cardiomyopathy (Wang et al., 2025). BMAL1 deficiency causes metabolic inflexibility characterized by sustained fatty acid oxidation and impaired glucose utilization via disrupted AKT/GSK3β signaling, accompanied by defective BNIP3-dependent mitophagy, IP3R-induced calcium overload, suppressed respiratory complex activities, and increased ROS generation (Li et al., 2020). These combined metabolic and mitochondrial defects drive cardiomyocyte apoptosis, diminish contractile force, and activate necrotic signaling, thereby promoting myocardial injury and HF development (Del Re et al., 2019) (Fig. 2, orange pathway).

Beyond metabolic regulation, the circadian clock also modulates the cardiac response to acute hypoxic stress. Ruan et al. recently revealed that BMAL1, typically dimerized with CLOCK, forms an alternative heterodimer with HIF2A during hypoxia (Ruan et al., 2025). This builds on earlier work demonstrating that cardiomyocyte-specific HIF2A induces amphiregulin (AREG) to dampen myocardial IR injury (Koeppen et al., 2018), and that Adora2b-elicited PER2 stabilization promotes a HIF-dependent metabolic switch crucial for myocardial adaptation to ischemia (Eckle et al., 2012). This BMAL1-HIF2A complex binds hybrid HRE/E-box motifs and rhythmically induces AREG, a cardioprotective factor whose ablation abolishes time-of-day-dependent cardiac protection. BMAL1 stabilizes HIF2A protein via reduced ubiquitination, amplifying hypoxic responses (Ruan et al., 2025). This pathway explains the larger infarct size and poorer prognosis observed in morning-onset MI (corresponding to low AREG phase). Pharmacologically, the HIF2A stabilizer Vadadustat enhances BMAL1-HIF2A binding and cardioprotection when administered at Zeitgeber time 8:00 (ZT8), while Nobiletin or recombinant AREG rescues damage at ZT20 (Ruan et al., 2025). These findings provide proof-of-principle evidence that targeting the circadian clock can mitigate myocardial injury in a time-dependent manner (Fig. 2, blue pathway).

Clinical investigations reveal specialized circadian disruption signatures in HF (Crnko et al., 2023). In HFrEF patients, peripheral cardiac clocks remain functional, evidenced by robust diurnal oscillation of cardiac troponin T, whereas central clock output is impaired, with dampened melatonin amplitude and blunted cortisol rhythm, manifesting as non-dipping blood pressure/heart rate in >77% patients (Crnko et al., 2023). This central-peripheral dichotomy is corroborated across species (nocturnal mice, diurnal zebrafish), showing preserved core clock gene phase relationships in failing hearts despite systemic disease (Crnko et al., 2023). Together, these findings support the broader concept that circadian control of cardiac metabolism, hypoxic adaptation, and injury tolerance contributes to myocardial resilience, while circadian disruption may weaken metabolic flexibility and repair capacity.

Collectively, these findings demonstrate that circadian control of cardiac metabolism contributes to myocardial energetic reserve and injury tolerance. When normal temporal organization is disrupted, cardiomyocytes may lose metabolic flexibility, mitochondrial efficiency, and the ability to match energy production with contractile and repair demands. Acute ischemic vulnerability may therefore be greater when endogenous clock-dependent protective programs are relatively weak, whereas chronic HF may involve sustained central–peripheral circadian misalignment rather than a single time-of-day defect. This supports a cautious rationale for rhythm-restoring and time-aware metabolic interventions to preserve mitochondrial function and myocardial resilience.

### 3.3 Fibrosis

#### 3.3.1 Cellular drivers of fibrosis

Pathological cardiac remodeling (encompassing fibrosis, hypertrophy, and ventricular stiffening) is the structural consequence of sustained myocardial injury and inflammation. The molecular circadian clock critically coordinates the cellular effectors of this process. BMAL1 may act as a potent inhibitor of fibrosis. Decreased BMAL1 levels in aged or injured cardiac fibroblasts correlate with elevated S100A4 and impaired scar containment after infarction (Sato et al., 2017). This is consistent with the broader observation that fibroblast mobilization and wound healing are under circadian control, with the timing of injury influencing fibrotic outcomes (Hoyle et al., 2017). REV-ERBα curbs myofibroblast differentiation and collagen secretion in cardiac fibroblasts. Loss of REV-ERBα function impairs this inhibitory brake on fibrosis (Luo et al., 2022) (Fig. 2, purple pathway). *In vivo*, pharmacological activation of REV-ERBα further prevents cardiac hypertrophy and reduces established fibrosis in mouse models of HF (Zhang et al., 2017).

Macrophage pro-fibrotic signaling is also clock-controlled. BMAL1 and REV-ERB direct infiltrating macrophages towards pro-reparative M2-like polarization and pro-fibrotic cytokine secretion (Nguyen et al., 2013; Wang et al., 2022). REV-ERBα mediates the circadian gating of macrophage innate immunity; its deletion abolishes diurnal rhythmicity of inflammatory cytokines including IL-6 (Gibbs et al., 2012). Conversely, LPS-induced miR-155 targets the *Bmal1* 3′-UTR, suppressing BMAL1 and reinforcing NF-κB-driven inflammation (Curtis et al., 2015). Systemically, myeloid *Bmal1* deficiency disrupts diurnal Ly6C^hi^ monocyte oscillation, increasing constitutive tissue infiltration (Nguyen et al., 2013), and monocyte circadian disruption is directly linked to cardiac inflammation and fibrosis in cardiorenal syndrome (Yoshida et al., 2021). This dysregulation disrupts the balance between beneficial healing and excessive scarring. Maladaptive crosstalk between dysregulated immune cells and stromal cells sustains fibrotic cascades and SMC phenotypic switching (Heusch et al., 2014; Yoshida et al., 2021). Pericyte loss or damage fosters vascular instability and may enable trans-differentiation into pro-fibrotic myofibroblasts (Alex et al., 2023). Although circadian links in pericytes require further investigation, their vulnerability intersects with the clock-regulated health of neighboring ECs and smooth muscle.

Together, these observations suggest that fibrosis after cardiac injury may be phase-dependent, particularly when fibroblast mobilization and monocyte recruitment coincide with weakened clock-dependent anti-fibrotic restraint. However, this timing model remains provisional because acute and chronic cardiac remodeling after HF may follow distinct circadian kinetics.

#### 3.3.2 Cellular perpetuators of fibrosis

Adipocyte biology is increasingly recognized in HF and exhibits circadian dependencies. Circadian disruption, including adipocyte-specific *Bmal1* deletion (Paschos et al., 2012), promotes adipocyte hypertrophy, adipose inflammation, fibrosis, and dysregulated adipokine secretion (Packer, 2018; Xiong et al., 2021; Yepuri et al., 2021). In obesity, visceral adipose tissue displays disrupted clock gene expression correlating with aberrant inflammatory factor secretion (Maury et al., 2021). These secreted factors signal to cardiomyocytes, fibroblasts, and immune cells, promoting cardiac fibrosis, hypertrophy, and inflammation (Yepuri et al., 2021) (Fig. 2, purple pathway). Moreover, the vasoregulatory role of perivascular adipose tissue (PVAT) via renin-angiotensin system signaling is modulated by adipocyte BMAL1, linking circadian disruption to hypertension and hemodynamic stress in HF (Chang et al., 2018). Adipocyte AMPK gates diurnal rhythmicity of circulating metabolites (Chen et al., 2025), suggesting that adipose clock dysfunction may compromise inter-organ metabolic coordination in HF.

Beyond cell-autonomous effects, clock disruption amplifies maladaptive intercellular communication, fueling pathological remodeling. In ECs, BMAL1 deficiency promotes ROS-induced Endothelial-to-Mesenchymal Transition (EndoMT)-like changes through BMP signaling, consistent with the recognized role of EndoMT as a contributor to fibrotic diseases (Zhu et al., 2018). A murine chronic jet lag model (serial 8 h phase advance every 4 days for 3 months) recapitulates HFpEF (Che et al., 2024). Downregulated cardiac *Clock* impairs sGC-cGMP-PKG1 signaling, reducing PKG1 and titin expression, and inducing cardiomyocyte hypertrophy and interstitial fibrosis (Che et al., 2024). *Clock* knockdown in isolated cardiomyocytes recapitulates these effects cell-autonomously (Che et al., 2024). These findings are consistent with reduced PKG activity in human HFpEF myocardium and its correlation with nitrosative/oxidative stress and metabolic comorbidities (van Heerebeek et al., 2012). The sGC stimulator riociguat attenuates the increases in left ventricular mass and collagen deposition and improves diastolic function in this model (Che et al., 2024). The overall consequence is profound: unchecked fibrogenic activity drives excessive ECM deposition, resulting in increased myocardial stiffness, impaired ventricular relaxation (diastolic dysfunction), and progressive adverse left ventricular remodeling (Mia et al., 2022) (Fig. 2, purple pathway). These are core features that drive progression to symptomatic HF and decompensation.

Vascular and adipose contributions to cardiac remodeling likely reflect cumulative chronic circadian misalignment rather than a discrete time-of-day vulnerability. Persistent disruption of adipocyte and endothelial clocks may impair PVAT-dependent vascular regulation, adipokine rhythms, endothelial identity, and microvascular stability, converging to promote adipose inflammation, EndoMT-like changes, fibrosis, hypertrophy, and diastolic dysfunction. Thus, vascular and adipose clock dysfunction may serve as a chronic remodeling amplifier through maladaptive paracrine, endocrine, and microvascular signaling.

### 3.4 Heart arrhythmia

The heart's core function of generating electrical impulses and converting them into mechanical contractions, exhibits clear circadian dependence. Disruption of this circadian control increases arrhythmia susceptibility and impairs contractile performance, contributing to HF morbidity and mortality (Delisle et al., 2024; Mia et al., 2020).

At the electrophysiological level, core clock proteins regulate the expression of key cardiac ion channels and their transcriptional regulators, shaping daily variation in heart rate, repolarization, and arrhythmia susceptibility (Mia et al., 2020; Schroder et al., 2021). Multiple cardiac ion channel genes, including *Scn5a* (Nav1.5), *Kcnh2* (hERG), and *Kcnd2* (Kv4.2), exhibit circadian oscillation in expression, providing a molecular basis for circadian variation in cardiac electrophysiology (Delisle et al., 2024). BMAL1 coordinates cardiac electrical stability, driving sex-specific circadian ion channel regulation and time-of-day differences in arrhythmia susceptibility (Delisle et al., 2026). Cardiomyocyte-specific *Bmal1* deletion (CBK) disrupts circadian ion-channel expression, downregulating key repolarizing potassium channels such as *Kcnh2* (Schroder et al., 2015) and creating an arrhythmogenic substrate of slower heart rate, RR- and QT-interval prolongation, and increased susceptibility to ventricular arrhythmia including life-threatening ventricular fibrillation (Gottlieb et al., 2021). This laboratory finding mirrors the well-established diurnal peak in malignant arrhythmias (Fig. 2, green pathway) observed clinically and underscores the role of BMAL1 in preserving electrical homeostasis. Metabolic disturbances from clock disruption (Section 3.2) also impair ion channel function and excitability through altered substrate availability and reactive oxygen species accumulation, further elevating arrhythmia risk (El Jamal et al., 2023).

These observations suggest that arrhythmia susceptibility may be increased around the rest-to-active transition, which corresponds to the early morning period in humans. During this window, phase-dependent ion-channel regulation may converge with increased metabolic demand, oxidative stress and repolarization instability to promote abnormal excitability. This phase-based interpretation is consistent with the morning predominance of malignant arrhythmias and post-MI ventricular tachycardia, but the optimal timing of chronotherapeutic interventions remains to be tested.

### 3.5 Contractile dysfunction

Circadian disruption can impair cardiac contractile performance. BMAL1 deficiency compromises cardiomyocyte contractile force and beating regularity, accompanied by abnormal Ca^2+^ handling, disorganized myofilaments and disrupted sarcomeric architecture (Li et al., 2020). Mechanistically, cardiomyocyte *Bmal1* deletion alters Ca^2+^-handling gene expression, including *Cacna1c*, *Ryr2* and *Casq2* (Hayter et al., 2021), and circadian-clock regulation of the PKA–phospholamban–SERCA2a reuptake pathway provides an additional link (Zhang et al., 2020). In HFpEF specifically, the primary Ca^2+^ defect is impaired diastolic calcium clearance rather than systolic release failure, distinguishing it mechanistically from HFrEF (Frisk et al., 2021). Whether clock disruption selectively exacerbates this diastolic Ca^2+^ clearance deficit remains to be determined. Ischemia–reperfusion disturbs cardiac clock-gene oscillations, transiently uncoupling clock-controlled metabolic and repair programs (Martino & Young, 2015). Recent evidence reveals an adaptive mechanism whereby BMAL1 under ischemic stress associates with HIF2A to confer time-dependent cardioprotection (Ruan et al., 2025). The metabolic deficits from clock disruption create an energetic deficit that undermines the ATP demands of systolic contraction and diastolic calcium reuptake (Zhang et al., 2020).

Because cardiomyocyte circadian-clock programs modulate Ca^2+^ handling and energy metabolism, contractile vulnerability may be heightened after ischemic insults at the rest-to-active transition (the early morning in humans). Ischemia-induced disruption of BMAL1-dependent programs may therefore weaken the temporal coordination among Ca^2+^ handling, energy metabolism, and stress-response pathways. Whether HFpEF-related diastolic Ca^2+^ clearance defects are selectively amplified during this window remains unresolved, but overlap with the arrhythmia-prone period suggests that morning-onset injury may impair electrical and mechanical recovery in parallel.

The net effect of circadian disruption on cardiac electromechanical function is profoundly deleterious: unstable rhythms, diminished cardiac output, increased morbidity, reduced functional capacity, and heightened risk of sudden cardiac death (Fig. 2, green pathway).

### 3.6 Circadian and sleep-wake influences on cardiovascular rhythmicity

An important issue in interpreting time-of-day variation in cardiovascular events is the extent to which these patterns reflect endogenous circadian regulation or sleep-wake-dependent changes in behavior and autonomic tone. The circadian clock is a cell-autonomous molecular oscillator that can sustain an approximately 24 h rhythm under constant conditions, whereas the sleep-wake cycle is a behavioral state shaped by both circadian timing and homeostatic sleep pressure (Takahashi, 2017). These processes are closely coupled in daily life, and changes in posture, activity, feeding and sympathetic tone across sleep-wake transitions may amplify cardiovascular rhythmicity. Nevertheless, tissue-specific studies demonstrate a direct cardiac contribution. Cardiomyocyte clock disruption attenuates or abolishes the time-of-day dependence of myocardial ischemia-reperfusion tolerance in mice (Durgan et al., 2010). Cardiac glucocorticoid receptor signaling further links the daily corticosteroid surge to rhythmic ion-channel transcription and intrinsic ventricular arrhythmia susceptibility, while largely preserving core clock-gene rhythmicity in cardiomyocytes (Tikhomirov et al., 2024). Together, these findings support an interactive model in which cardiac-local clocks integrate sleep-wake-dependent autonomic, hormonal and behavioral signals. Distinguishing their relative contributions in humans will require controlled protocols that separate endogenous circadian phase from sleep, activity, posture and meal timing.

## 4 Cell type-specific functions of the cardiac circadian clock in HF

This section examines how key circadian clock components function within distinct cardiac cell populations and how their disruption contributes to HF pathophysiology. BMAL1/CLOCK and REV-ERBα/β functions across major cardiac cell types are summarized in Table 3.

**Table 3:** BMAL1/CLOCK and REV-ERBα/β functions across major cardiac cell types.

Cell type	Clock component	Target/function	Key reference(s)	Evidence
**Cardiomyocyte**	BMAL1	BNIP3-dependent mitophagy	Li et al. (2020)	Established
	BMAL1	*Cacna1c* *Ryr2* *Casq2*	Hayter et al., 2021; Zhang et al., 2020	Established
	BMAL1	*Kcnh2* *Scn5a*	Schroder et al., 2015; Delisle et al., 2026	Established
	BMAL1	HIF2A heterodimer → AREG cardioprotection	Ruan et al. (2025)	Established
	BMAL1	AKT/GSK3β signaling; metabolic flexibility	Li et al. (2020)	Established
	BMAL1	Diastolic function, inflammatory resolution (cKO)	Ingle et al. (2015)	Established
	CLOCK	sGC-cGMP-PKG1 pathway (HFpEF)	Che et al. (2024)	Established
	REV-ERBα	CDKN1A/p21 repression → I/R vulnerability	Montaigne et al. (2018)	Established
	REV-ERBα/β	E4BP4 → NAMPT-NAD^+^ biosynthesis	Dierickx et al. (2022)	Established
	REV-ERBα/β	*Cpt1a* *Ech1*	Dierickx et al. (2022)	Established
	REV-ERB	Diurnal metabolite and lipid rhythms	Song et al. (2022)	Established
	REV-ERBα	MEF2 suppression → anti-hypertrophy/anti-fibrosis	Zhang et al. (2017)	Established
**Endothelial cell**	BMAL1	NF-κB suppression → anti-inflammatory	Xie et al. (2020)	Established
	BMAL1	Anti-EndoMT (BMP signaling)	Zhu et al. (2018)	Established
	BMAL1	*CCNA1* *CDK1*	Astone et al. (2023)	Established
	BMAL1	eNOS coupling (GTPCH-1, DHFR)	Anea et al. (2012)	Established
	PER2	HIF1A-dependent glycolytic adaptation	Eckle et al. (2012)	Established
	PER2	Endothelial barrier protection (light-mediated)	Oyama et al. (2019)	Established
	CLOCK/BMAL1	Adhesion molecule rhythmicity (ICAM-1, VCAM-1)	Scheiermann et al. (2012)	Established
**Vascular SMC**	BMAL1	Vasoconstrictor responsiveness (ROCK2)	Xie et al. (2015)	Established
	BMAL1	Anti-atherosclerotic (NRF2, Bcl-2)	Lin et al. (2022)	Established
	RORα	ROCK2-dependent vascular contractility rhythm	Saito et al. (2013)	Established
**Macrophage**	BMAL1	Mitochondrial metabolism; anti-inflammatory	Alexander et al. (2020)	Established
	BMAL1	NRF2-dependent IL-1β suppression	Early et al. (2018)	Established
	REV-ERBα	IL-6 and Ccl2 suppression	Gibbs et al., 2012; Sato et al., 2014	Established
	BMAL1	miR-155-mediated inflammatory suppression	Curtis et al. (2015)	Established
**Monocyte**	BMAL1	Ly6C(hi) monocyte diurnal oscillation	Nguyen et al. (2013)	Established
	BMAL1	GPR68-mediated cardiac infiltration (CKD)	Yoshida et al. (2021)	Established
**Neutrophil**	BMAL1	CXCL2-CXCR2 diurnal aging	Adrover et al. (2019)	Established
	BMAL1	CXCL12-CXCR4 circadian checkpoint	Aroca-Crevillen et al. (2026)	Established
**Fibroblast**	BMAL1	S100A4/p21 suppression	Sato et al. (2017)	Proposed
	REV-ERBα/β	Myofibroblast differentiation restraint	Luo et al. (2022)	Established
	REV-ERBα	TBPL1-integrinβ1-focal adhesion (pulmonary)	Cunningham et al. (2020)	Proposed
**Adipocyte**	BMAL1	Angiotensinogen regulation in PVAT	Chang et al. (2018)	Established
	BMAL1	Whole-body energy timing	Paschos et al. (2012)	Established
	BMAL1	NF-κB-associated inflammatory remodeling	Maury et al. (2021)	Proposed
	REV-ERBα	Adipose expansion and inflammation (obesity)	Hunter et al. (2021)	Established
**Pericyte**	—	Microvascular stability (circadian evidence limited)	Alex et al., 2023; Simmonds et al., 2024	Proposed

Note: Established: direct evidence from cell-type-specific genetic models or well-validated mechanistic studies in cardiac systems. Proposed: indirect evidence, correlative data, or evidence from non-cardiac systems. SMC, smooth muscle cell; PVAT, perivascular adipose tissue; EndoMT, endothelial-to-mesenchymal transition; cKO, conditional knockout; CKD, chronic kidney disease.

### 4.1 Cardiomyocytes

Cardiomyocytes harbor the best-characterized cell-autonomous circadian clock in the heart. The functional roles of BMAL1 in cardiomyocyte metabolism, mitochondrial quality control, and Ca^2+^ handling are detailed in Sections 3.2, 3.5. At the transcriptional level, BMAL1 directly regulates *Bnip3* (mitophagy), *Cacna1c*, *Ryr2* and *Casq2* (Ca^2+^ handling and electrical stability) (Hayter et al., 2021; Li et al., 2020; Zhang et al., 2020). Under hypoxic stress, BMAL1 forms a heterodimer with HIF2A to induce AREG, conferring circadian-phase-dependent cardioprotection (Ruan et al., 2025). The REV-ERBα–CDKN1A/p21 signaling pathway underlying the time-of-day variation in myocardial ischemia-reperfusion tolerance is discussed in Section 3.1 (Montaigne et al., 2018).

REV-ERBα/β provide context-dependent regulation. Genetic deletion of both receptors causes dilated cardiomyopathy and premature death through derepression of E4BP4, impairing NAMPT-dependent NAD^+^ biosynthesis and suppressing fatty-acid oxidation genes (Dierickx et al., 2022). Song et al. further showed that cardiac Rev-erb coordinates diurnal metabolite and lipid rhythms, with obesity-associated fatty-acid supply partially rescuing cardiac dysfunction (Song et al., 2022). Yet REV-ERB activity is not uniformly protective: during ischemic stress, elevated REV-ERBα represses CDKN1A/p21 and increases myocardial vulnerability, whereas REV-ERBα antagonism reduces injury (Montaigne et al., 2018). Conversely, pharmacological REV-ERBα activation suppresses MEF2-associated pathological gene programs and attenuates hypertrophy and fibrosis in pressure-overload HF models (Zhang et al., 2017). Thus, REV-ERB effects are phase-, stress- and context-dependent rather than uniformly protective or detrimental.

Genetic disruption of core clock components further supports a causal link between cardiomyocyte timekeeping and HF-relevant remodeling (El Jamal et al., 2023). Cardiomyocyte-specific *Bmal1* deletion produces age-dependent diastolic dysfunction, cardiac hypertrophy, interstitial and endocardial fibrosis, extracellular-matrix remodeling and impaired inflammatory resolution (Ingle et al., 2015). Systemic *Bmal1* deficiency similarly leads to age-dependent dilated cardiomyopathy, and *Clock*^*Δ19*^ mutation causes cardiac hypertrophy, fibrosis and impaired systolic performance in aged mice (Lefta et al., 2012). Together with the Rev-erbα/β loss-of-function models discussed above, these findings indicate that disruption of the cardiomyocyte clock does not produce a single stereotyped phenotype; rather, distinct clock nodes bias the heart toward metabolic failure, adverse remodeling or contractile dysfunction depending on the genetic lesion and stress context.

### 4.2 Endothelial cells

Endothelial clocks shape vascular functions that are relevant to HF progression, including inflammatory tone, endothelial identity and angiogenic repair. In human aortic endothelial cells and atherosclerosis models, BMAL1 downregulation enhances oxidative stress and NF-κB signaling, supporting an anti-inflammatory role for endothelial BMAL1 (Xie et al., 2020). BMAL1 loss also promotes ROS- and BMP-dependent EndoMT-like changes, linking endothelial clock disruption to vascular remodeling programs associated with plaque vulnerability (Zhu et al., 2018). In ischemic angiogenesis models, circadian disruption or CRY1/2 deficiency impairs perfusion recovery and endothelial proliferative, migratory and tube-forming capacity, accompanied by decreased HOXC5 and increased WEE1 expression (Tsuzuki et al., 2021). More directly, endothelial BMAL1 supports developmental and pathological angiogenesis by binding the promoters of CCNA1 and CDK1 and sustaining endothelial cell-cycle progression (Astone et al., 2023).

Endothelial clock components also regulate vasodilatory and ischemia-adaptive programs. In Bmal1-deficient vessels, impaired rhythmic expression of GTPCH-1 and DHFR disrupts biopterin homeostasis, uncouples eNOS and shifts endothelial signaling from NO production toward superoxide generation (Anea et al., 2012). Consistently, *Per2* mutation impairs endothelium-dependent aortic relaxation through reduced NO and vasodilatory prostaglandin signaling and increased COX-1-derived vasoconstrictor activity (Viswambharan et al., 2007). In the ischemic heart, PER2 further promotes HIF1A-dependent glycolytic adaptation and cardioprotection downstream of ADORA2B signaling (Eckle et al., 2012). This PER2-dependent ischemic program was later linked more directly to the endothelium, as endothelial *Per2* deficiency abolished intense-light-mediated cardioprotection and endothelial barrier protection during myocardial ischemia–reperfusion injury (Oyama et al., 2019).

Beyond NO signaling, vascular circadian programs also organize immune–endothelial interactions. Transcriptomic profiling of mouse aorta identified approximately 330 circadian transcripts enriched for vascular integrity, protein dynamics and injury-response pathways (Rudic et al., 2005). Functionally, leukocyte trafficking is gated by time-of-day-dependent migratory cues: tissue- and lineage-specific oscillations in pro-migratory factors guide leukocyte homing, and adrenergic inputs further entrain rhythmic recruitment to tissues (Scheiermann et al., 2012). Thus, the endothelial clock temporally regulates inflammatory cell entry rather than passively responding to inflammation. Endothelial clock disruption may therefore amplify HF-relevant microvascular inflammation by weakening NO-dependent protection, impairing repair, and mistiming leukocyte recruitment.

### 4.3 Smooth muscle cells

In vascular smooth muscle cells, the circadian clock primarily gates vascular tone and remodeling rather than acting as a direct HF effector. The RORα–ROCK2 pathway generates an intrinsic rhythm of vascular contractility by coordinating ROCK2 expression, myosin light-chain phosphorylation and smooth-muscle Ca^2+^ sensitization (Saito et al., 2013). Consistently, smooth-muscle-specific *Bmal1* deletion abolishes time-of-day variation in vasoconstrictor responses to K^+^, phenylephrine and serotonin, and disrupts blood-pressure rhythmicity through altered ROCK2-dependent contractile signaling (Xie et al., 2015). Beyond vasomotor control, VSMC-specific Bmal1 disruption worsens carotid atherosclerotic lesions by promoting RAC1-dependent VSMC migration, monocyte transmigration and oxidative stress while impairing NRF2- and *Bcl-2-*dependent antioxidant-survival programs (Lin et al., 2022). Together, these findings position the SMC clock as a regulator of vascular tone, inflammatory cell entry and lesion stability—processes that can amplify HF-relevant hemodynamic and microvascular stress.

Given that SMC BMAL1 regulates time-of-day vasoconstrictor responsiveness (Xie et al., 2015), vascular tone appears especially sensitive to clock disruption during phases when these rhythmic outputs are normally engaged. In pathological VSMCs, attenuation or phase distortion of this program may expose a substrate characterized by increased ROS production, migratory signaling, monocyte transmigration, and impaired NRF2/Bcl-2-dependent antioxidant-survival responses (Lin et al., 2022). Whether analogous clock-dependent vascular vulnerability contributes to the morning vasoconstrictor surge and cardiac-vascular injury in humans remains to be directly tested.

Although direct evidence for a cell-autonomous circadian clock in cardiac pericytes remains limited, pericyte dysfunction may intersect with endothelial and SMC clock programs by destabilizing the microvascular niche after myocardial ischemia and in HFpEF-relevant diastolic dysfunction models (Simmonds et al., 2024). The contribution of pericytes to cardiac pathology, notably through their impairment, functional loss, and potential acquisition of fibrosis-associated phenotypes (Alex et al., 2023), may contribute to fibrosis and microvascular instability in HF, although pericyte-autonomous circadian clock evidence remains limited compared with ECs or SMCs.

### 4.4 Cardiac immune cells

Immune cells harbor intrinsic circadian clocks that temporally organize leukocyte trafficking, cytokine production and redox-dependent inflammatory responses (Scheiermann et al., 2018). This temporal regulation is directly relevant to HF, in which unresolved inflammation contributes to adverse remodeling across both reduced- and preserved-ejection-fraction phenotypes (Alcaide et al., 2024). Among clock components, BMAL1 and REV-ERBα provide two well-characterized immune checkpoints: myeloid *Bmal1* regulates the diurnal oscillation and tissue recruitment of Ly6C^hi^ inflammatory monocytes (Nguyen et al., 2013), whereas REV-ERBα gates macrophage inflammatory cytokine output, including endotoxin-induced IL-6 responses (Gibbs et al., 2012). BMAL1 also restrains macrophage IL-1β production through NRF2-dependent redox control, linking circadian disruption to inflammasome-prone inflammatory states (Early et al., 2018). These mechanisms provide the rationale for considering neutrophils, monocytes and macrophages as time-sensitive contributors to cardiac injury, repair and remodeling.

#### 4.4.1 Neutrophils

Neutrophils are among the most time-sensitive immune effectors in cardiac injury. A neutrophil-intrinsic timer coordinates diurnal aging through BMAL1-dependent CXCL2-CXCR2 signaling, whereas CXCR4 antagonizes this aging program and promotes vascular protection (Adrover et al., 2019). In myocardial infarction models, the timing of ischemic onset determines cardiac neutrophil recruitment: injury at ZT13 produces greater CXCR2-dependent neutrophil infiltration than injury at ZT5, resulting in larger infarcts and worse cardiac function; limiting neutrophil recruitment at this phase reduces injury and improves repair (Schloss et al., 2016). Recent work identified a CXCL12-CXCR4 circadian checkpoint that restrains neutrophil-driven tissue damage by relocating neutrophils within the wound core and sparing adjacent host cells from apoptosis. Pharmacological CXCR4 activation protects against myocardial and vascular injury in preclinical models (Aroca-Crevillen et al., 2026). Together, these studies indicate that neutrophils can either amplify or limit post-ischemic injury depending on the circadian state of CXCR2-CXCR4 signaling, providing a mechanistic basis for neutrophil-targeted chronotherapy while underscoring the need for HF-specific validation.

#### 4.4.2 Macrophages and monocytes

Macrophage and monocyte clocks shape HF-relevant inflammation through two linked processes: inflammatory tone within macrophages and rhythmic recruitment of circulating monocytes. In macrophages, BMAL1 supports mitochondrial metabolism under inflammatory stress and restrains redox-amplified cytokine production; loss of *Bmal1* impairs mitochondrial fitness and enhances inflammatory activation in metabolically stressed macrophages (Alexander et al., 2020), while BMAL1-dependent NRF2 regulation limits ROS accumulation and IL-1β production (Early et al., 2018). The roles of REV-ERBα in gating macrophage IL-6 responses and Ccl2 expression are discussed in Section 3.3.1 (Gibbs et al., 2012; Sato et al., 2014).

At the monocyte level, the circadian regulation of Ly6C^hi^ monocyte oscillation and trafficking is discussed in Section 3.3.1 (Nguyen et al., 2013). More directly linking monocyte circadian machinery to cardiac remodeling, CKD-induced clock disruption increases GPR68 expression in circulating monocytes, promoting their cardiac infiltration and exacerbating inflammation and fibrosis in cardiorenal syndrome models (Yoshida et al., 2021). Together, these findings support a model in which macrophage clocks tune inflammatory responsiveness, whereas monocyte clocks gate cellular supply to injured or remodeling myocardium. Whether timed suppression of macrophage inflammatory signaling or monocyte recruitment improves HF outcomes remains an untested therapeutic question.

### 4.5 Cardiac fibroblasts

Cardiac fibroblasts mediate extracellular-matrix remodeling and fibrotic scar formation, and emerging evidence links their activation state to circadian clock components. Direct cardiac-fibroblast evidence is still limited, but available data implicate BMAL1 and REV-ERB as clock-linked restraints on fibroblast activation (Section 3.3.1; Luo et al., 2022; Sato et al., 2017). Supportive evidence from non-cardiac fibroblastic systems further shows that REV-ERBα restrains myofibroblast activation and pulmonary fibrosis through TBPL1–integrinβ1–focal-adhesion remodeling (Cunningham et al., 2020). Together, these findings suggest that fibroblast clocks may shape fibrotic remodeling by coordinating quiescence, migration and matrix-producing activation, although direct temporal mapping in cardiac fibroblasts during chronic HF and post-infarction scar formation remains lacking.

### 4.6 Cardiac adipocytes

Adipocytes, especially those in perivascular and epicardial adipose depots, connect circadian timing with metabolic and vascular signals that are relevant to HF. Adipocyte-specific deletion of *Bmal1* causes obesity, shifts the daily rhythm of food intake and reduces circulating polyunsaturated fatty acids, indicating that the adipocyte clock contributes to whole-body energy timing rather than only local lipid storage (Paschos et al., 2012). This metabolic role extends to vascular regulation through PVAT. Local PVAT Bmal1 rhythmically regulates angiotensinogen expression, and the resulting Ang II signal acts on adjacent smooth muscle cells to control resting phase vascular tone and blood pressure (Chang et al., 2018). In obesity, adipose clock dysfunction is linked to inflammatory transcriptional remodeling. In human omental fat, BMAL1 binding is redistributed near NF-κB-associated regulatory regions, altering genes involved in metabolic inflammation and adipose tissue remodeling (Maury et al., 2021). Together, these findings suggest that adipocyte clocks may influence HF-relevant cardiometabolic stress through metabolic, inflammatory and vasoactive outputs.

Adipose remodeling may further affect the heart through endocrine and local paracrine signals. Chronic circadian shift in mice induces adipocyte hypertrophy, macrophage infiltration, extracellular matrix remodeling, adipose inflammation and fibrosis, together with impaired insulin signaling (Xiong et al., 2021). In patients with obesity and HFpEF, epicardial adipose tissue has been proposed as a mediator of the effects of systemic inflammation and metabolic dysfunction on the myocardium, but this clinical link should not be interpreted as direct evidence for a clock-dependent mechanism (Packer, 2018). REV-ERBα adds further complexity because its adipocyte function depends on metabolic state. Adipocyte *Rev-erbα* deletion has limited effects under basal conditions, but during obesity it alters white adipose tissue expansion and protects against obesity-related inflammation, fibrosis and insulin resistance (Hunter et al., 2021). In brown adipose tissue, human studies show a glucose-responsive thermogenic rhythm associated with UCP1, GLUT4, and REV-ERBα expression, and lower glycemic variability (Lee et al., 2016). These findings support an indirect model in which adipocyte clock programs influence HF-relevant remodeling through systemic metabolism, adipose inflammation and local adipose signals, while direct evidence in HF remains limited.

### 4.7 Intercellular coordination of cardiac circadian clocks

The heart functions as a multicellular system in which circadian clocks in different cell types interact to maintain homeostasis. While each major cardiac cell type expresses core clock genes, emerging evidence suggests that their rhythmic outputs are not independent but coordinated through intercellular signaling. Litvinukova et al. mapped the cellular composition of the adult human heart using single-nucleus RNA sequencing, identifying cardiomyocytes, fibroblasts, endothelial cells, smooth muscle cells and immune populations as key constituents (Litvinukova et al., 2020). Although this atlas was generated from a single time point, it provides the cellular framework necessary to interpret how rhythmic signals may propagate across compartments.

Heart-specific *Bmal1* deletion is associated with increased p21, S100A4, CD206, and TNF-α expression in myocardial stromal fibroblasts, consistent with a non-cell-autonomous relationship between cardiac clock disruption and stromal activation (Sato et al., 2017). The mediating signals and their cellular source remain undefined. By contrast, REV-ERB activity has been examined directly in cardiac fibroblasts. Combined loss of REV-ERBα and REV-ERBβ reduces fibroblast viability and proliferation while increasing migration and myofibroblast activation, supporting a fibroblast-autonomous role for these nuclear receptors in maintaining stromal homeostasis (Luo et al., 2022). Thus, current evidence supports both tissue-level associations and cell-autonomous fibroblast mechanisms, but does not yet establish phase-dependent bidirectional signaling between cardiomyocytes and fibroblasts.

Endothelial cells regulate immune cell trafficking in a time-dependent manner. Nguyen et al. showed that myeloid *Bmal1* controls the diurnal oscillation of Ly6C^hi^ inflammatory monocytes in circulation (Nguyen et al., 2013). Scheiermann et al. established that adrenergic signals entrain rhythmic leukocyte homing, and that endothelial cells express adhesion molecules such as ICAM-1 rhythmically (Scheiermann et al., 2012). In a chronic kidney disease model, disruption of clock-gene expression in circulating monocytes increases GPR68 expression and promotes cardiac infiltration, inflammation, and fibrosis (Yoshida et al., 2021). These findings identify complementary temporal checkpoints in circulating immune cells and the vascular interface, although their coordination in primary HF remains incompletely defined.

Perivascular adipose tissue provides a direct example of local circadian signaling within the vascular wall. BMAL1 regulates angiotensinogen and local angiotensin II production in perivascular adipose tissue, thereby modulating adjacent vascular smooth muscle contraction and resting phase blood pressure (Chang et al., 2018). By comparison, adipocyte specific Bmal1 deletion alters feeding rhythms, circulating lipid signals and body weight, supporting a broader role for adipose clocks in systemic metabolic timing rather than direct adipose to myocardial communication (Paschos et al., 2012).

HF may also weaken coordination between systemic timing cues and peripheral oscillators. In patients with HFrEF, melatonin and cortisol rhythms are dampened while circulating cardiac troponin T remains rhythmic. Preserved core clock gene rhythms in the heart, kidney and liver have been demonstrated in complementary animal HF models rather than human cardiac tissue (Crnko et al., 2023). These findings raise the possibility of impaired central peripheral coordination, but direct measurements of tissue phase relationships in human HF are lacking. Nobiletin and timed feeding can modify clock amplitude or peripheral rhythmicity in metabolic models (He et al., 2016; Manella et al., 2021), but whether either intervention restores intercellular temporal coordination in HF remains unknown. Together, current evidence provides a framework for interpreting the convergent injury, inflammatory and fibrotic pathways shown in Fig. 2, while direct mapping of phase dependent cell to cell signaling in HF remains a priority.

### 4.8 Single-cell and spatial omics approaches to cardiac circadian biology

The cardiac cell atlases introduced in Section 1, including the adult human heart snRNA-seq resource (Litvinukova et al., 2020) and the murine Tabula Muris Senis aging atlas (Tabula Muris, 2020), provide a census of 11 major cardiac cell types and their canonical marker genes. However, these atlases were not designed with repeated sampling across defined circadian phases, and therefore cannot distinguish cell-autonomous circadian transcriptional rhythms from diurnal shifts in tissue composition. This distinction is functionally important: an apparent oscillation in pro-inflammatory gene expression could reflect either a genuine circadian rhythm within resident macrophages or a rhythmic influx of circulating Ly6C^hi^ monocytes that constitutively express inflammatory factors (Nguyen et al., 2013).

Time-resolved single-cell and single-nucleus RNA sequencing with biological replication across circadian phases could separate changes in cell state from changes in cellular composition. Integration with single-nucleus chromatin accessibility profiling could further identify cell-specific regulatory elements and test whether the cardiomyocyte REV-ERB, E4BP4 and NAMPT pathway established in mouse hearts operates across cardiac cell populations and disease states (Dierickx et al., 2022). Cross-species phase alignment should be based on biological phase rather than direct conversion of murine Zeitgeber time to human clock time, because core clock components show an approximately 12 h phase difference between nocturnal mice and diurnal primates despite broadly conserved relative phase relationships (Mure et al., 2018).

Time-resolved spatial transcriptomics could localize temporal programs to vascular, fibrotic, infarct-border and remote myocardial niches. When combined with inducible lineage tracing and cell-specific genetic perturbation, such as cardiomyocyte *Bmal1* deletion, these approaches could distinguish primary clock effects from secondary responses in neighboring cells (Ingle et al., 2015). Lineage tracing could further determine whether temporal changes in fibroblast or immune cell states arise within resident populations or through recruitment and replacement. A time- and space-resolved atlas of the failing heart would therefore provide a testable framework for reconstructing temporal intercellular networks. At present, proposed links among endothelial recruitment signals, infiltrating monocytes, and S100A4-expressing fibroblasts should be viewed as hypotheses rather than established circadian circuits (Sato et al., 2017; Yoshida et al., 2021).

## 5 Translational perspectives: targeting the clock for HF therapy

### 5.1 Pathogenic model: circadian disruption as a multicellular amplifier of HF progression

The evidence reviewed in Sections 3, 4 supports a model in which circadian clock dysfunction amplifies HF progression across the cardiac multicellular network (Fig. 3(A)). In cardiomyocytes, BMAL1 loss weakens mitochondrial quality control, metabolic homeostasis, contractile function, and electrical stability (Sections 3.2, 3.5, 4.1). In endothelial and smooth muscle cells, clock disruption impairs angiogenic repair, inflammatory control, and vascular tone regulation (Sections 4.2, 4.3). In immune cells, BMAL1 and REV-ERBα regulate inflammatory cell supply and cytokine tone (Sections 3.3.1, 4.4). In fibroblasts and adipocytes, available evidence is more indirect, but clock programs influence fibroblast activation, adipose inflammation, and PVAT vascular signaling (Sections 3.3, 4.5, 4.6). Together, these cell-type-specific findings indicate that circadian clock dysfunction is not a single upstream cause of HF but a timing defect that weakens metabolic, electrical, inflammatory, vascular, and fibrotic checkpoints across the cardiac multicellular network.

**Fig. 3:**
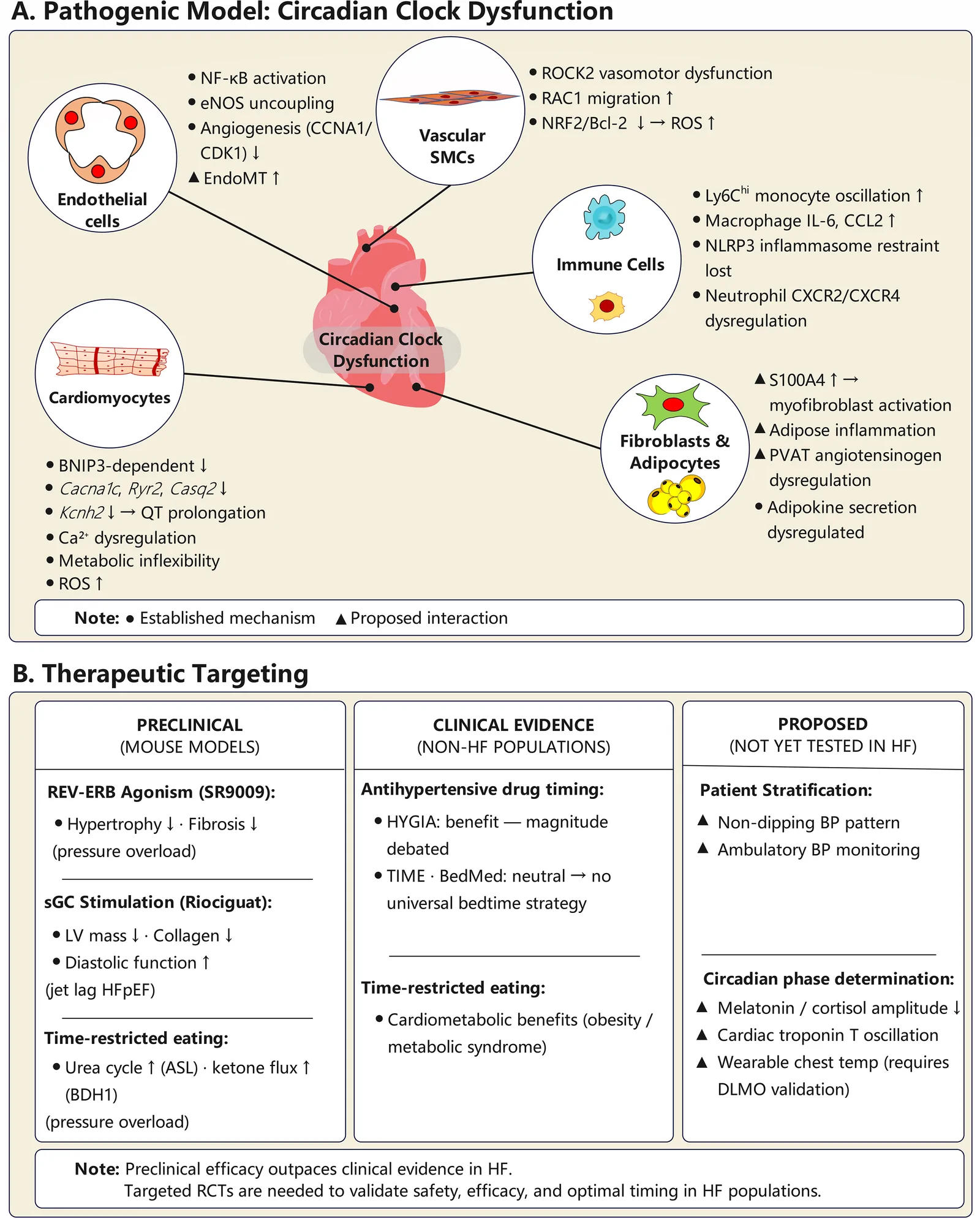
**Circadian clock dysfunction and therapeutic targeting of the circadian clock in heart failure**. (A) Pathogenic Model: Circadian Clock Dysfunction. Central-peripheral clock decoupling is characterized by dampened central hormonal output (melatonin, cortisol) with preserved peripheral cardiac clocks. In cardiomyocytes, BMAL1 loss impairs mitophagy, metabolic flexibility, and electrical stability, leading to QT prolongation, Ca^2+^ dysregulation, and ROS accumulation. In endothelial cells, BMAL1 deficiency activates NF-κB signaling, uncouples eNOS, and impairs angiogenic capacity; EndoMT-like changes are a proposed mechanism (triangle bullet in the figure). In vascular SMCs, Bmal1 disruption abolishes time-of-day vasoconstrictor responsiveness, promotes migration, and impairs antioxidant programs. In immune cells, myeloid Bmal1 regulates monocyte diurnal oscillation, and loss of inflammasome restraint increases inflammatory cytokine output. In fibroblasts and adipocytes, established mechanisms include adipokine secretion defects; proposed mechanisms include PVAT angiotensinogen dysregulation, myofibroblast activation, and adipose inflammation. Solid bullets denote established mechanisms; triangle bullets denote proposed interactions. (B) Therapeutic Targeting, organized by level of supporting evidence. Tier 1 (preclinical, mouse models): REV-ERB agonism attenuates hypertrophy and fibrosis in pressure-overload models, with context-dependent cardiac effects; sGC stimulation improves diastolic function and reduces left ventricular mass and collagen in chronically jet-lagged mice; time-restricted eating (TRE) protects against pressure-overload HF via urea cycle activation and ketone flux. Tier 2 (clinical evidence, non-HF populations): bedtime antihypertensive dosing has shown benefit in one large trial but not in subsequent pragmatic randomized trials; TRE improves cardiometabolic measures in obesity and metabolic syndrome, with no direct evidence in established HF. Tier 3 (proposed, not yet tested in HF): non-dipping blood pressure and ambulatory monitoring as stratification tools; melatonin, cortisol and cardiac troponin T rhythms as candidate phase readouts; wearable chest-surface temperature monitoring, which requires validation against established phase markers. Overall, preclinical efficacy outpaces clinical evidence in HF; targeted randomized trials are needed.

REV-ERB signaling adds further complexity because its effects differ by cell type and disease context: it maintains NAMPT-dependent NAD^+^ biosynthesis in cardiomyocytes (Section 4.1; Dierickx et al., 2022) yet promotes Ccl2-driven monocyte recruitment when lost in macrophages (Section 4.4; Sato et al., 2014). These divergent effects argue against a simple model in which activating or inhibiting one clock component is uniformly beneficial. Instead, circadian disruption may reinforce several HF pathways simultaneously: myocardial injury promotes inflammation, inflammation contributes to fibrosis and microvascular dysfunction, and these changes further impair perfusion, increase myocardial stiffness, and weaken repair capacity (Heusch et al., 2014). This multicellular, feed-forward view is more consistent with the evidence reviewed above than a single linear pathway from clock dysfunction to HF.

### 5.2 Clinical evidence and the limits of chronotherapy

Antihypertensive medication timing provides the most extensively tested example of cardiovascular chronotherapy, yet current evidence does not support a uniform bedtime dosing strategy for all patients. Early observational studies and the Hygia Chronotherapy Trial reported that bedtime administration of at least one antihypertensive medication improved sleep time blood pressure control and was associated with fewer cardiovascular events (Hermida et al., 2020) (Fig. 3(B)). The large reported benefit, however, prompted debate regarding its magnitude and interpretation. Subsequent pragmatic randomized trials did not reproduce a general cardiovascular advantage. In the TIME study, evening administration of usual antihypertensive therapy did not improve major cardiovascular outcomes compared with morning dosing (Mackenzie et al., 2022). BedMed similarly found that bedtime dosing was safe but did not reduce death or major cardiovascular events in adults with hypertension in primary care (Garrison et al., 2025).

Together, these trials do not support a universal bedtime dosing strategy. Nocturnal hypertension and non-dipping blood pressure remain plausible stratification variables, but whether they predict benefit from treatment timing has not been established prospectively in HF. Future trials should therefore prespecify circadian phenotypes, incorporate ambulatory blood pressure monitoring, and assess HF-specific efficacy and safety outcomes rather than extrapolating from general hypertensive populations.

### 5.3 Challenges in translation and personalized implementation

Translating chronotherapeutic findings from nocturnal rodents to humans requires more than a fixed 12 h conversion of dosing time. Comparative transcriptomics shows that the peak phases of core clock genes differ by approximately 12 h between nocturnal mice and diurnal baboons, while the identity and phase of many rhythmic transcripts differ across tissues and species (Mure et al., 2018). Experimental timing should therefore be aligned to biological phase, including rest-activity and feeding cycles, rather than transferred directly from murine Zeitgeber time to human clock time.

Personalized chronotherapy also depends on reliable measures of circadian phase. In patients with HFrEF, melatonin and cortisol retain 24 h rhythmicity but show reduced amplitude and variation, whereas cardiac troponin T remains significantly rhythmic. Phase relationships among core clock genes are also preserved in complementary mouse and zebrafish HF models (Crnko et al., 2023). These measures provide candidate readouts of circadian organization, but their value for phase estimation or treatment stratification in HF remains unvalidated.

Wearable monitoring may provide a scalable complement to hormonal sampling. Continuous chest-surface temperature monitoring can characterize individual circadian parameters under daily-life conditions (Huang et al., 2021).

However, wearable-derived phase estimates require further validation against established phase markers such as dim-light melatonin onset before they can guide treatment timing in HF. Chronotherapy should therefore be tested with stratification by individual circadian phenotype rather than applied according to clock time alone.

### 5.4 Phenotype- and etiology-specific circadian mechanisms in HF

HF comprises heterogeneous phenotypes and etiologic contexts, and the contribution of circadian disruption is unlikely to be uniform. HFrEF and HFpEF are defined by ejection fraction, whereas ischemic, hypertensive and obesity-related HF reflect overlapping disease backgrounds. Current evidence is strongest for cardiomyocyte intrinsic mechanisms and remains more indirect for vascular and adipose pathways.

In HFrEF, cardiomyocyte clocks regulate processes relevant to systolic function. Cardiomyocyte clock disruption attenuates the time-of-day dependence of ischemia reperfusion injury, establishing a direct role in acute myocardial stress responses rather than chronic HFrEF itself (Durgan et al., 2010). Chronic cardiac loss of REV-ERBα and REV-ERBβ abolishes diurnal rhythms in myocardial metabolic gene expression and leads to progressive dilated cardiomyopathy with systolic dysfunction in mice (Song et al., 2022). Together, these studies link impaired cardiac timekeeping with metabolic and contractile abnormalities relevant to reduced EF states, although a common HFrEF specific clock mechanism has not been established.

In HFpEF, circadian disruption may interact with metabolic and diastolic abnormalities. Repeated circadian phase shifts induce cardiac hypertrophy, fibrosis and diastolic dysfunction with preserved ejection fraction in mice, accompanied by reduced Clock, sGC, cGMP and PKG1 signaling (Che et al., 2024). Pharmacological stimulation of sGC attenuates these changes (Che et al., 2024). Reduced myocardial PKG activity and increased cardiomyocyte resting tension have also been reported in human HFpEF (van Heerebeek et al., 2012). These findings support cGMP and PKG signaling as one potential link between circadian disruption and diastolic dysfunction, particularly in cardiometabolic HFpEF, but do not define a universal HFpEF mechanism.

Ischemic HF introduces a distinct temporal vulnerability linked to acute injury timing. Montaigne et al. showed that perioperative myocardial injury was greater after morning than afternoon aortic valve surgery, with experimental evidence implicating REV-ERBα in this time dependent vulnerability (Montaigne et al., 2018). These findings support time-dependent myocardial vulnerability but should not be extrapolated directly to chronic ischemic HF.

Hypertension related HF may involve circadian regulation upstream of the myocardium. Smooth muscle Bmal1 contributes to blood pressure and vascular tone rhythms (Xie et al., 2015), while Bmal1 in perivascular adipose tissue regulates angiotensinogen and local angiotensin II signaling to adjacent vascular smooth muscle cells (Chang et al., 2018). Disruption of these pathways alters blood pressure level and rhythmicity, although a direct causal link to hypertensive cardiac remodeling or HF has not been demonstrated.

Adipose clocks may similarly influence the metabolic background of obesity-related HF. Adipocyte specific *Bmal1* deletion alters feeding rhythms, circulating lipid signals and energy balance in mice (Paschos et al., 2012). Human obesity is also associated with altered clock regulation in visceral adipose tissue and increased inflammatory transcriptional activity (Maury et al., 2021). These findings connect adipose clock disruption with systemic metabolic stress, but direct circadian signaling from adipose tissue to the failing myocardium remains poorly defined.

Together, circadian mechanisms appear to differ across HF phenotypes and disease contexts, although direct evidence remains limited. Future trials should account for HF phenotype, etiology and circadian status when evaluating timed interventions.

### 5.5 Time-restricted eating: a chrono-nutrition strategy for cardiometabolic health

Beyond pharmacological strategies, time-restricted eating (TRE) provides a non-pharmacological way to align daily feeding behavior with circadian organization. TRE limits food intake to a defined 6 to 12 h window without necessarily reducing caloric intake. In this Review, TRE should be framed as a strategy for organizing peripheral metabolic rhythms and cardiometabolic risk, rather than as an established HF therapy. Earlier work summarized the broader links among fasting, circadian rhythms and healthy lifespan (Longo & Panda, 2016), while a TRE-focused review framed clock-modulated checkpoints in tissue function and noted that direct evidence in established HF remains limited (Li, 2022). This framework nonetheless supports discussing TRE as a candidate rhythm-aligned intervention for HF-relevant metabolic and inflammatory stress.

Clinical studies support cardiometabolic benefits of TRE, but direct evidence in established HF remains limited. Trials in prediabetes, metabolic syndrome and obesity report improvements in insulin sensitivity, body weight, blood pressure or lipid profiles (Jamshed et al., 2022; Wilkinson et al., 2020). Early evidence in patients with prior myocardial infarction suggests that short-term early TRE may reduce systemic inflammatory markers and modulate innate immune readouts (Los et al., 2026). Related studies in metabolically at-risk populations also suggest possible benefits for myocardial enzyme profiles, glycemic control or body composition, particularly when TRE is combined with other lifestyle interventions (Haganes et al., 2022). Thus, TRE should be viewed as a cardiometabolic intervention that warrants HF specific testing rather than as a proven HF therapy.

The HF-relevant mechanisms of TRE likely involve both peripheral clock organization and metabolic remodeling. The heart appears less responsive to feeding time than the liver and several metabolic tissues, suggesting that some cardiac effects of TRE may arise indirectly through inter-organ rhythmic coordination rather than direct myocardial clock resetting (Li, 2022). Consistent with this view, the liver clock coordinates rhythmicity of peripheral tissues in response to feeding, especially under nutritional challenge (Manella et al., 2021). TRE may also engage metabolic pathways relevant to HF. Intermittent time restricted feeding supports BDH1-dependent ketone flux and metabolic remodeling in skeletal and cardiac muscle (Williams et al., 2024), while time restricted feeding protects against pressure-overload-induced HF in mice through increased cardiac amino acid utilization and ASL-dependent urea cycle activation (Tan et al., 2024). Adipocyte thermogenesis may further contribute to obesity-related metabolic benefits of time restricted feeding (Hepler et al., 2022). Together, these findings suggest that TRE may influence HF-relevant stress through inter organ timing, substrate handling and adipose metabolism, but the relative contribution of these pathways in human HF remains unresolved.

Several limitations should temper the interpretation of TRE studies. Benefits observed in some trials may reflect reduced caloric intake rather than circadian realignment. In adults with obesity, TRE combined with caloric restriction was not superior to caloric restriction alone, and the TREAT trial found no clear advantage of 16:8 TRE over usual meal timing for weight loss or cardiometabolic markers (Liu et al., 2022; Lowe et al., 2020). The ChronoFast trial further showed that intended isocaloric TRE shifted circadian markers without improving insulin sensitivity or conventional cardiometabolic risk factors (Peters et al., 2025). Although broader intermittent fasting evidence also remains uncertain, it is not specific to TRE (Allaf et al., 2021). Therefore, HF-specific trials are needed to determine whether TRE directly improves HF outcomes or mainly acts through comorbid metabolic risk.

## 6 Conclusion

This review summarizes evidence that the circadian clock system involving multiple cell-autonomous clocks is an essential component of heart functions related to the pathophysiology of HF. We propose that HF is not only a state of structural and functional impairment but also in part, a state of disordered timekeeping across multiple cardiac cell types. This framework supports the potential of targeting circadian components, which remains to be tested in HF trials. Restoring temporal coherence through refined pharmacological strategies and/or timed lifestyle interventions may offer a complementary approach to heart health.

## CRediT authorship contribution statement

**Shuting Cheng:** Writing – original draft, Investigation, Conceptualization. **Runchao Zhou:** Writing – original draft, Investigation, Conceptualization. **Zhihui Zhang:** Writing – review & editing, Writing – original draft, Investigation, Funding acquisition, Conceptualization. **Min-Dian Li:** Writing – review & editing, Writing – original draft, Supervision, Funding acquisition, Conceptualization.

## Declaration of competing interest

The authors declare that they have no known competing financial interests or personal relationships that could have appeared to influence the work reported in this paper.
